# In Silico Modeling of Acoustic Microbubble Dynamics to Optimize Blood‐Brain Barrier Disruption for Safe Drug Delivery in Alzheimer’s Treatment

**DOI:** 10.1002/alz70859_097606

**Published:** 2025-12-25

**Authors:** Folasewa Maryam Abdulsalam, Orit Shefi Neuro‐Engineering Research Lab

**Affiliations:** ^1^ Bar‐Ilan University, Ramat‐Gan, Tel‐Aviv Israel

## Abstract

**Background:**

The Blood‐Brain Barrier (BBB) is a significant challenge in delivering therapeutic agents to the brain, particularly in the treatment of Alzheimer’s disease. Acoustic‐driven microbubbles have emerged as a potential method for transient BBB disruption, allowing targeted drug delivery.

**Method:**

This ongoing research employs an in‐silico model developed in COMSOL Multiphysics to simulate the interaction of microbubbles with acoustic waves and their effects on BBB permeability. The current stage involves assigning material properties to brain capillaries and microbubbles and setting boundary conditions to capture fluid‐structure interactions under acoustic pressure.

**Result:**

The result aims to determine the conditions under which microbubble oscillations can effectively open the BBB while minimizing tissue damage. Preliminary simulations are focused on refining model parameters to align with physiological constraints.

**Conclusion:**

While results are pending, this study is expected to contribute to a better understanding of the parameters needed for safe and controlled BBB disruption, advancing drug delivery strategies for Alzheimer’s treatment.

